# Surface Modification of Biochar to Prepare Environmentally Friendly Electrochemical Biosensors for Detection of Cardiac Troponin T

**DOI:** 10.1021/acsomega.5c02113

**Published:** 2025-06-09

**Authors:** Aline Macedo Faria, Rafael Aparecido Ciola Amoresi, Larissa Bach-Toledo, Juan Andrés, Talita Mazon

**Affiliations:** † 193232 Centro de Tecnologia da Informação Renato Archer (CTI) − Ministério da Ciência, Tecnologia e Inovação (MCTI), Rod. D. Pedro I, KM 143.6, 13069-901 Campinas, SP, Brazil; ‡ Department of Analytical and Physical Chemistry, 16748 University Jaume I (UJI), Av. Vicent Sos Baynat, 12071 Castelló, Spain

## Abstract

Driven by the need to investigate enhanced biosensing properties alongside the development of low-toxicity, economical, eco-friendly, and sustainable materials, this work explores the functionalization of biochar—a carbon-based material—and its subsequent anchoring onto the working electrode for the detection of cardiac troponin T (cTnT). Here, we discuss the interaction between biochar and glutaraldehyde at various concentrations, aiming to elucidate the relationship between the formation of full acetals and hemiacetals. It first allows an understanding of the cross-link reactions between glutaraldehyde and biochar and then better anchoring with the Cystamine-Au working electrode of a printed circuit board (PCB). These aspects improve the biosensor’s stability and the cTnT antibody’s specific adsorption. Electrochemical, AFM, Fluorescence Confocal, SEM, and FTIR analyses were employed to identify the optimal glutaraldehyde concentration that maximizes hemiacetal group formation, critical for stability to the electrode and the specific adsorption of the antibody. The resulting label-free, direct electrochemical cTnT immunosensor showed high sensitivity and selectivity, with a detection capacity of 0.01–5.00 ng·mL^–1^ and a Limit of Detection (LOD) of 0.003 ng·mL^–1^, as determined by cyclic voltammetry. An additional advantage is the reusability of the PCB, which can be recycled at least twice by replacing the biochar layer on the working electrode, assigning an environmentally friendly characteristic to the immunoassay.

## Graphical abstract

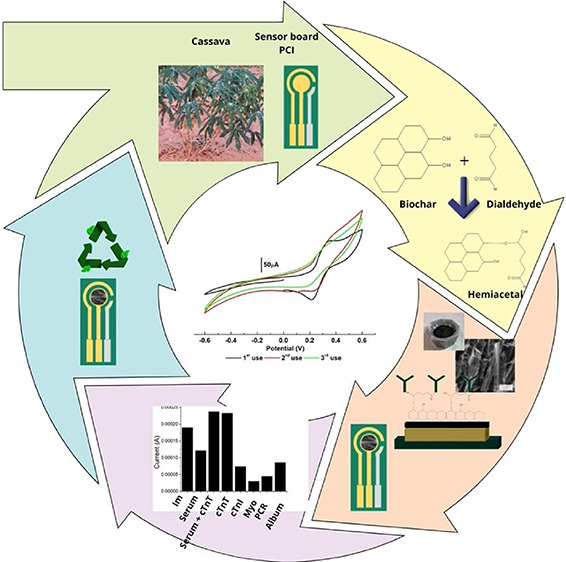

## 1 Introduction

Label-free immunosensors, a type of biosensor, detect the formation of an immunocomplex between a specific target analyte (antigen) and the corresponding biomarker (antibody), leading to the generation of a measurable signal given by a transducer.^1–4^ Unlike traditional diagnostic techniques, such as microscopy, colony counting, enzyme-linked immunosorbent assay (ELISA), or Polymerase Chain Reaction (PCR) tests, label-free immunosensors do not require labeling agents. They offer significant advantages, including real-time detection, reduced complexity, and, most importantly, lower costs, making them ideal for rapid and sensitive biomolecular analysis.^5–7^ Immunosensors, classified based on their transduction mode, include optical, microgravimetric, thermometric, and electrochemical responses.^8^ Among these, electrochemical immunosensors stand out for their sensitivity, selectivity, low sample volume, independence from sample turbidity, and, most importantly, cost-effectiveness.^9,10^ These electrochemical immunosensors can detect biomarkers in body biofluids, such as enzymes, proteins, and antibodies, acting directly in their detection.^11^

Several strategies have been employed to modify the immunosensor electrodes to enhance the signal response, improve the detection limit, and increase the sensor selectivity. Among the most prominent approaches are incorporating semiconductor oxide nanoparticles, metallic nanoparticles, and the formation of nanocomposites.^12–14^ These modifications are designed to improve electronic conductivity, facilitate the functionalization of the components, enhance the catalytic activity, and improve biocompatibility with the antibody attached, thereby enabling precise control over the properties of the immunosensor.^15^ Beyond the pursuit of improved analytical performance, there is a growing demand for developing low-toxicity, cost-effective, eco-friendly, sustainable, and biocompatible materials while also exhibiting synergy with electrochemical performance for applications across a wide range of diagnostic platforms.^16,17^ In this context, carbon-based materials, such as graphene and its derivatives, have attracted considerable attention. These materials offer excellent properties, including high charge mobility, large surface area, and ease of functionalization.^18–20^ However, current synthesis methods for these materials often fall short of achieving consistent stability and uniformity. Alternatively, developing graphene-based hybrid nanocomposites increases both the synthesis steps and the overall cost of the sensing device.^21^ A promising alternative to graphene technology is another carbonaceous material: biochar. Biochar is a green-synthesized material with a high carbon content, obtained through the pyrolysis of various types of biomass, such as corn stalks, grain waste, sugar cane bagasse, cassava, and others. Many of these are found in many countries and generate substantial waste at almost every production stage.^22,23^

Biochar has emerged as a promising biosensor application due to its high porosity, electrical conductivity, stable electrochemical performance, and low cost.^24^ Recent studies by Cancelliere et al. have demonstrated that biochar-based electrodes can surpass commercial graphene electrodes regarding current response and biomolecule immobilization efficiency.^25^ Their work also demonstrated superior electrochemical performance of the biochar-modified electrodes, regardless of the electroactive species used, and enhanced immobilization of interleukin, a pro-inflammatory cytokine associated with health disorders.^26,27^ In addition to these findings, other research groups have reported excellent biosensing performance of biochar-modified electrodes in detecting a wide range of biomolecules and microorganisms, including E. coli, SARS-CoV-2, Hantavirus, and glucose, enabling the potential of biochar in diverse applications such as medical diagnostics, environmental monitoring, and food safety.^28–31^ Therefore, the use of biochar, with its chemical structure rich in hydroxyl groups and high surface area, can offer multiple advantages for biosensor technology,^32–35^ including (i) providing a highly functional surface to immobilize biomolecules, (ii) enhancing surface kinetics and electrochemical performance, and (iii) increasing the hydrophilicity of the device, making it particularly suitable for applications such as biosensor devices that require capturing body fluids.^36^ Moreover, biochar plays an important role in waste valorization. It adds value to agro-industrial byproducts, supports sustainability initiatives, promotes a circular economy, and aids in carbon sequestration.^37,38^

When employing biochar in label-free biosensors to enhance sensitivity, two key challenges must be addressed: improving conductivity and preventing nonspecific adsorption of biomolecules on the electrode surface. While the first challenge—enhancing conductivity—has been constantly pursued, often through incorporating carbon nanotubes, carbon paste, semiconductors, or magnetic nanoparticles to amplify the electrochemical signal and, consequently, the sensitivity of biosensors,^33,34,39^ the second issue— nonspecific adsorption—remains relatively underexplored. Proteins, including antibodies commonly used in biosensors, consist of one or more amino acid residues that tend to adsorb nonselectively onto various surfaces, including biochar. Although the carboxylic groups present in biochar can facilitate the covalent attachment of proteins via amine linkages,^25,30,34^ nonspecific adsorption negatively impacts the signal-to-noise ratio and reduces biosensor sensitivity, compromising the accuracy and reliability of measurements.^40^ The most widely used method for specific protein immobilization onto carbonaceous surfaces involves activating carboxylic acid using 1-ethyl-3,3-dimethyl carbodiimide and *N*-hydroxysulfosuccinimide (EDC/Sulfo-NHS) [8]. However, this approach has several limitations, including instability in aqueous solution and strong dependence on electrostatic preconcentration, ionic strength, and pH. An alternative strategy involves the activation of surface amines using glutaraldehyde (Glut) to create aldehyde-functionalized surfaces that promote the specific adsorption of biomolecules.^41^ Although less commonly applied to carbonaceous materials, this strategy is simple and enables specific immobilization of proteins on different surfaces, such as gold nanoparticles and zinc nanorods.^42,43^ Previous studies have shown that Glut provides a greater attachment of streptavidin on amine-terminated self-assembled monolayers (SAMs) than EDC/NHS.^40^ Given these advantages, studying the interaction between glutaraldehyde and biochar to improve the stability of the electrode and the immobilization of proteins is worthwhile. It could help to develop biosensors with better performance, low cost, high sensitivity, eco-friendliness, and reusable.

Cardiac troponin T (cTnT) is a biomarker for diagnosing acute coronary syndromes and heart failure.^44,45^ Elevated cTnT levels above 0.1 ng·mL^–1^ can lead to myocardial damage and a range of complications, including death.^45,46^ The National Academy of Clinical Biochemistry emphasizes the need to rapidly detect cardiac biomarkers, such as cTnT, with a recommended turnaround time of less than 1 h. Rapid diagnosis is crucial for effective treatment and preventing patients outcomes.^47–50^ While conventional analyses struggle to meet this short time frame, point-of-care (POC) electrochemical biosensors as an alternative for easy, rapid, and sensitive cTnT detection are promising and should be further explored.

This study aims to develop a label-free electrochemical immunosensor with enhanced performance achieved by using biochar activated with glutaraldehyde to immobilize cardiac troponin T (cTnT) antibodies on the working electrode. Utilizing biochar activated with glutaraldehyde promotes the preparation of stable working electrodes and the specific adsorption of cardiac troponin T (cTnT) antibodies. Furthermore, incorporating biochar enhances sensor accuracy while contributing to sustainability by reducing the environmental impact. A printed circuit board (PCB) containing the three integrated electrodes was used as a bare-board sensor. Biochar with a high degree of graphitization was produced from the pyrolysis of cassava biomass. Different concentrations of glutaraldehyde were tested during the activation of the biochar surface, and the influence of the formation of hemiacetal and full acetal groups on the performance of the immunosensor is discussed. The immunosensor was assayed for reproducibility, repeatability, specificity, selectivity, calibration curve, and limit of detection. The immunosensor detected cTnT in serum in 20 min with high accuracy and sensitivity. The board sensor can be recycled and reused, assigning an environmentally friendly characteristic to the immunoassay.

## 2 Experimental Section

### 2.1 Preparation of Biochar from Cassava Biomass

The biochar used in this work is produced from cassava waste. The cassava species used in this study was Manihot esculenta Crantz, planted in the municipality of Echaporã, SP-Brazil, at latitude 22°15′, longitude 50°07′, and a height of 700 m above sea level. The plant was extracted from the soil to harvest the cassava roots. The harvest residue was the stem and the thin and thick branches of the cassava plant. The cassava waste was previously macerated in a mortar and pestle to obtain smaller granules. Following the biomass maceration in mortar, the biomass was put in an alumina crucible and heat-treated at 750 °C for 4 h in an inert atmosphere. A flowchart of the preparation of cassava to obtain biochar is shown in Figure 1a.

**Figure 1:**
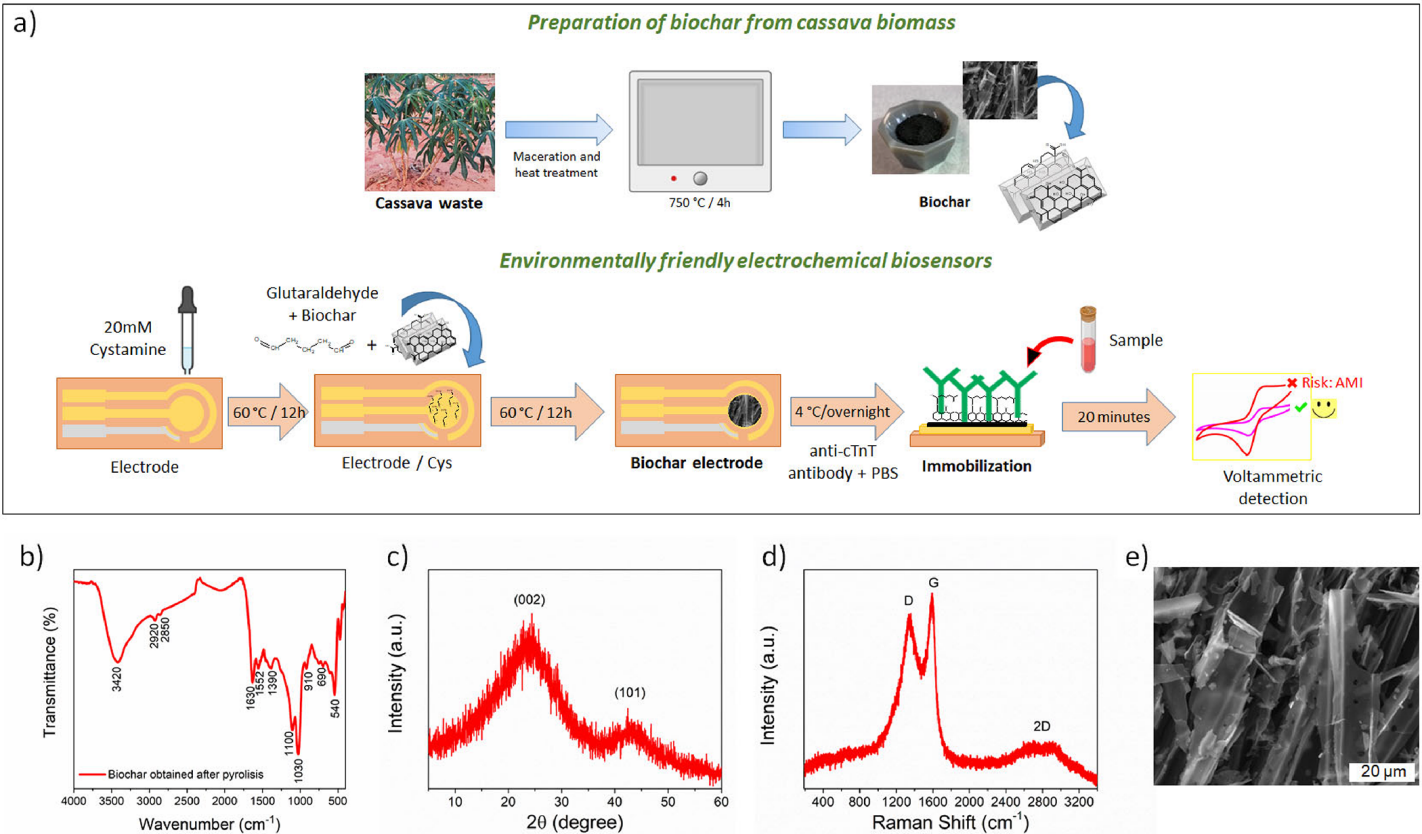
(a) Scheme for biosensor fabrication and (b) FTIR, (c) XRD, (d) Raman, and (e) SEM analyses of the biochar obtained after pyrolysis of cassava biomass.

### 2.2 Preparation of the Biochar Electrode

The bare board sensor was made on FR-4 (1.6 mm thick) sheets using Printed Circuit Board Technology (PCB). As previously described, it comprises one gold working, one gold counter electrode, and one silver/silver chloride (Ag/AgCl) reference electrode.^42^ The gold working electrode (Au WE) was incubated with 20 mM Cystamine dihydrochloride (Cys) (Alfa Aesar) at 60 °C for 12 h. Biochar from cassava biomass (0.01 g) was added to different glutaraldehyde (Glut) concentration solutions of 1.0–50%, mixed, and stirred overnight to prepare a homogeneous Glut-modified biochar paste. Glut-modified biochar paste was mixed with 5 wt % polyvinylidene difluoride (PVDF), deposited by drop-casting on the Cys-Au WE and incubated at 60 °C/12 h to promote the cross-linking with the amine-terminated monolayer.

### 2.3 Immobilization of the Anticardiac Troponin T Antibody

The anticardiac Troponin T antibody (Abcam (Cambridge, MA, USA)) was immobilized via 20 mM Cys and 2.5% Glut on the biochar film. Twenty microliters of anticardiac Troponin T antibody solution diluted in 0.1 M buffered phosphate saline (PBS) pH 7.4 at concentrations 1:5000 were added to the surface of the working electrode and incubated overnight at 4 °C in a moist chamber.

### 2.4 Electrochemical Measurements

The analytical responses of the immunosensor were evaluated by electrochemical measurements using cyclic voltammetry (CV) and differential pulse voltammetry (DPV). The assays were performed in the potential range from −0.6 to 0.6 V at the scan rate of 100 mV·s^–1^ recorded in a solution of 10 mmol·L^–1^ K_3_[Fe­(CN)_6_]/ K_4_[Fe­(CN)_6_] and 0.5 mol·L^–1^ NaNO_3_, whose pH is 6.0, as a mediator. Electrochemical impedance spectroscopy (EIS) was applied to investigate the processes on the surface of the biochar-coated electrode and compare them with those of the immunosensor. The measurements were performed at open circuit potential (OCP) in an amplitude of 10 mV and a frequency range from 0.1 Hz to 150 kHz, using [Fe­(CN)_6_]^−3/–4^ as a redox probe.

### 2.5 Characterization Methods

Samples were characterized by scanning electron microscopy (SEM), Mira 3 Tescan, with energy-dispersive X-ray spectroscopy (Brookfield), a confocal Raman model Horiba T64000 spectrometer with an exposure time of the 30 s, accumulation of 10 spectra, and LASER source of 633 nm, respectively, Fourier Transform Infrared Spectroscopy (FTIR) using a Thermo Scientific Smart iTR Nicolet iS10, coupled with the Attenuated Total Reflectance (ATR) smart accessory from Thermo Scientific, and BET method from nitrogen gas adsorption–desorption isotherms at 77 K using a ASAP 2010, Micromeritics. Atomic force microscopy analysis, EasyScan 2 (Nanosurf), was carried out in dynamic force no-contact mode; the scan size was set to 25 × 25 μm, with 1024 × 1024-pixel resolution. AFM Budget 150AI tips were used with a spring constant of 5 n/m. Images acquired by AFM were analyzed in Gwyddion by using specialized open-source image processing software. The immunosensors were evaluated by fluorescence intensity in an immunofluorescence confocal microscope, Leica, TCS SP5 II model, and a Duetta spectrometer (Fluorescence and Absorbance Spectrometer/Horiba Scientific). For these analyses, the sensors were incubated with Alexa Fluor 594 (Life Technologies) fluorescent secondary antibody in a 1:500 ratio for 1 h at room temperature.

### 2.6 Immunosensor Performance

The performance of the immunosensors was evaluated by reproducibility, repeatability, calibration curve, and limit of detection (LOD). The immunosensor was washed with PBS and then incubated with cardiac troponin T antigen in different concentrations (from 0.01 to 5.0 ng·mL^–1^) at room temperature, followed by washing with PBS buffer. 0.1 ng·mL^–1^ human cardiac troponin T antigen was incubated for 10, 20, and 30 min to find the best incubation time. All experiments were performed in a moist chamber. The evaluation was performed by CV analysis.

### 2.7 Specificity and Selectivity of the cTnT Immunosensor

Dot blot analysis is a traditional method used to assess the specificity of the cTnT immunosensor using recombinant Human cardiac Troponin I Protein (Abcam–Cambridge). First, a nitrocellulose membrane was prepared with dots (2 μL each) of different concentrations of recombinant Human cardiac Troponin I Protein (1.5 μg, 0.15 μg, 0.015 μg, 1.5 ng, and 0.15 ng). The membranes were allowed to dry for 30 min. After drying, the anti-cTnT antibody (1:1 000) was incubated in the membrane overnight at 4 °C. The blots were washed in Tris-buffered saline solution with Tween and incubated with horseradish peroxidase (HRP)-conjugated secondary antibody (Santa Cruz). Immunoreactivity dots were visualized by using the enhanced chemiluminescence method. For the selectivity study, dot blot analysis was performed using 2 μL of 150 ng·mL^–1^ recombinant molecules of cardiac Troponin I (cTnI), C Reactive Protein (CRP), and Myoglobin (Myo) dropped in a nitrocellulose membrane. These were then incubated with the anti-cTnT antibody, as previously described. The protein concentration of 150 ng·mL^–1^ was chosen due to the limit of detection of the dot blot technique.

Additionally, electrochemical assays were performed as described in section 2.4 by incubating the cTnT biosensors with serum, albumin, serum plus cTnT (0.1 ng·mL^–1^), cTnI (0.1 ng·mL^–1^), Myo (150 ng·mL^–1^), and CRP (0.8 μg·mL^–1^). The protein concentration was chosen based on the expected concentration in the human serum of a healthy patient.

### 2.8 Statistical Analysis

The reproducibility analysis of the sensors was calculated according to the coefficient of variation (CV) (eq 1):

CV=(S/X)×100 1

where (*S*) is the standard deviation and (*X*) is the average of the anodic current peak (*I*_pa_) performed on three different sensors of the assay. The analysis is considered reproducible when it presents a coefficient of variation equal to or less than 10%.

## 3 Results and Discussion

### 3.1 Biochar from Cassava: Morphological and Structural Characterization

FTIR spectroscopy evaluated the biochar obtained after pyrolysis of cassava biomass (Figure 1b). The spectrum revealed the presence of C–H, C=C, and C–C bonds, along with surface functional groups, including O–H and C–O. A broad band observed in the 3650–3200 cm^–1^ region is attributed to the O–H stretching vibration from hydroxyl groups and adsorbed water. This band appears weak due to the high heat treatment temperature during biochar production. Weak bands in the 2920–2850 cm^–1^ range correspond to Csp^2^–H stretching of the aromatic compounds/aliphatic groups, which are confirmed by the bands at 910 and 690 cm^–1^, associated with out-of-plane C–H deformation in aromatic compounds. The peaks observed at 1630 and 1550 cm^–1^ refer to the carbonyl group, C=O, and C=C in aromatic rings, respectively. A band in the region 1390 cm^–1^ is assigned to C–H stretching. The band at 1100 cm^–1^ corresponds to the C–O–C aliphatic stretching. The band at 1030 cm^–1^ corresponds to C–O ether vibration.^51–53^

XRD and RAMAN analyses were used to characterize the degree of graphitization of the biochar. Two diffraction peaks around 23° and 44° shown in the XRD diffractogram are assigned to typical (002) and (101) reflections of graphitic carbon (JCPDS number 41-1487), respectively (Figure 1c). The presence of the two broad peaks suggests a disordered structure and a graphitic lattice, which should contribute to higher electrical conductivity.^54^ RAMAN spectrum showed the D, G, and 2D bands that confirm the existence of graphitic carbon domains (Figure 1d). The D band at 1330 cm^–1^ is related to structural defects in the hexagonal framework associated with sp^3^-hybridized carbons.^55,56^ The G band at 1559 cm^–1^ corresponds to the graphitic structure and comes from the E_2g_ symmetry vibration mode of sp^2^ carbons.^57^ The 2D band at around 2680 cm^–1^ is a second order of the D band, and its broader shape consisting of two components indicates a multilayer graphite structure.^58^ The intensity D/G ratio was 0.87, which is related to the high graphitization degree of the biochar (Figure 1d). RAMAN result corroborates with FTIR and XRD results and proves the obtention of the biochar with a graphitized structure from cassava biomass. SEM analyses were performed to characterize the microstructure of the biochar as obtained (Figures 1e and S1). The biochar showed the morphology of microfibers with the presence of porous fibers on their surface. This porous structure could help in the diffusion of the electrolyte and improve the transport of the electron. The BET surface area of the biochar, determined by the Brunauer–Emmett–Teller (BET) method, was found to be 111 m^2^/g. The average pore diameter of biochar, calculated using the 4 V/A method, was approximately 2.2 nm. The nitrogen gas adsorption/desorption isotherm and corresponding pore size distribution are shown in Figure S2.

### 3.2 Biochar Sensor Characterization

Determining the optimal Glut-to-biochar ratio is essential for preparing a homogeneous and stable film on the Cys-Au WE and efficiently immobilizing the anticardiac Troponin T antibody. SEM, FTIR, and electrochemical analyses were performed to identify the most suitable conditions for preparing the Glu/biochar electrode based on glutaraldehyde concentration, film homogeneity, and electrochemical performance. These results are presented in Figures S3, 2, and 3, respectively. Figure S3 shows SEM images illustrating the homogeneous surface of the films prepared with two different glutaraldehyde concentrations: 2.5% (Glut-2.5%) and 50.0% (Glut-50%). The obtained films exhibited a rough surface characterized by micrometric fibers. In Figure S3a, prepared with Glut-2.5%, the biochar fibers appear dispersed, with the electrode surface only partially covered due to the low amount of glutaraldehyde used. In contrast, Figures S3b–d (Glut-50%) reveal a denser distribution of fibers compared to the previous sample covering all electrode surfaces. This results in a surface with pores of varying sizes and hollow channels. Given that the surface of the biochar particles is primarily composed of aromatic structures and hydroxyl groups, there is a strong affinity for glutaraldehyde molecules. This interaction leads to both specific and nonspecific covalent bonds, enhancing the stability between the biochar microfibers and the electrode surface.^59^

**Figure 2:**
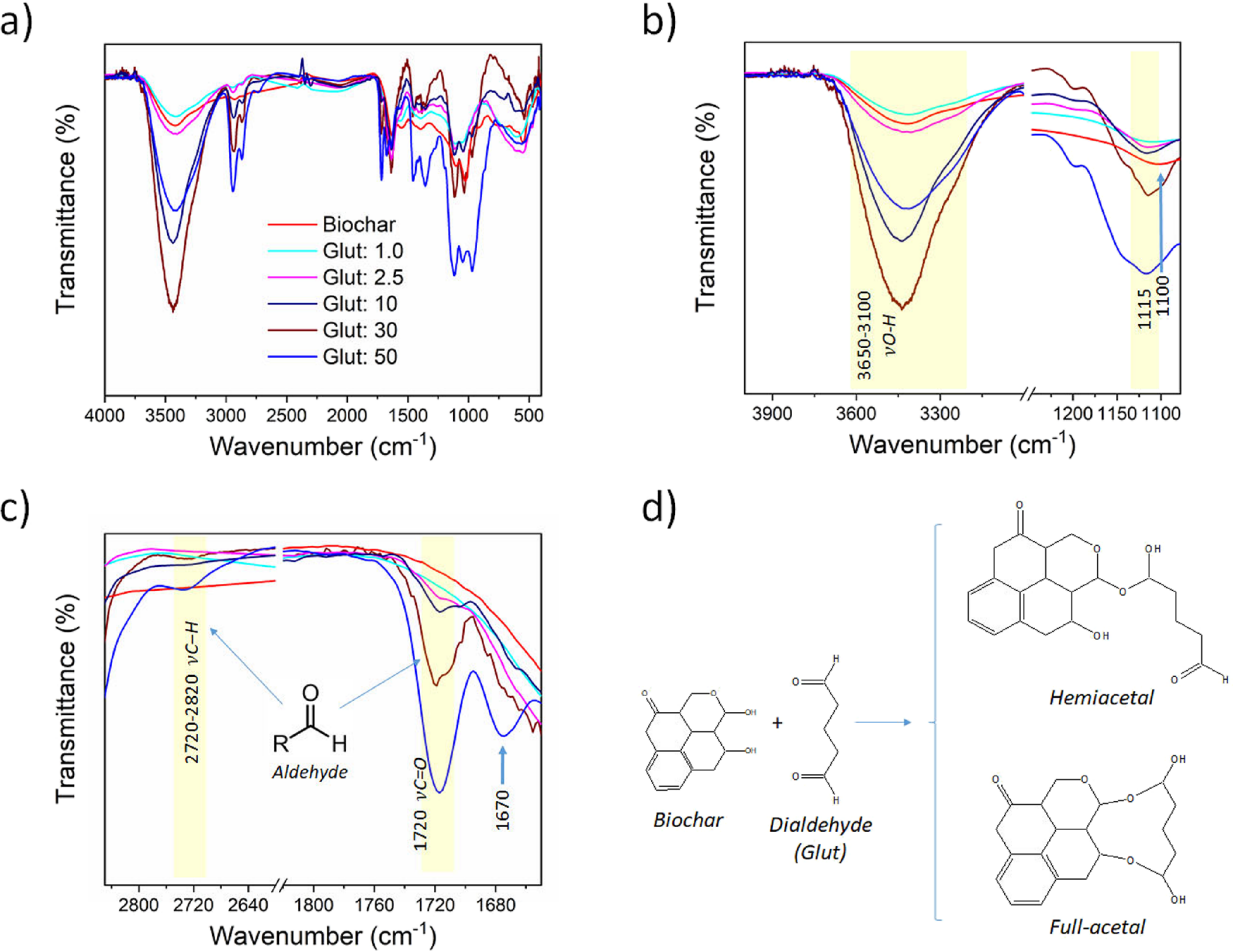
(a-c) FTIR analysis of the biochar:Glut system at different dilutions of glutaraldehyde, illustrating in (a) the full spectrum, (b) the OH and C–O–C vibrational regions, and (c) the aldehyde regions and the different carbonyl environments. In (d), a schematic diagram between the biochar surface and glutaraldehyde illustrates the hemiacetal and full acetal conditions.

**Figure 3:**
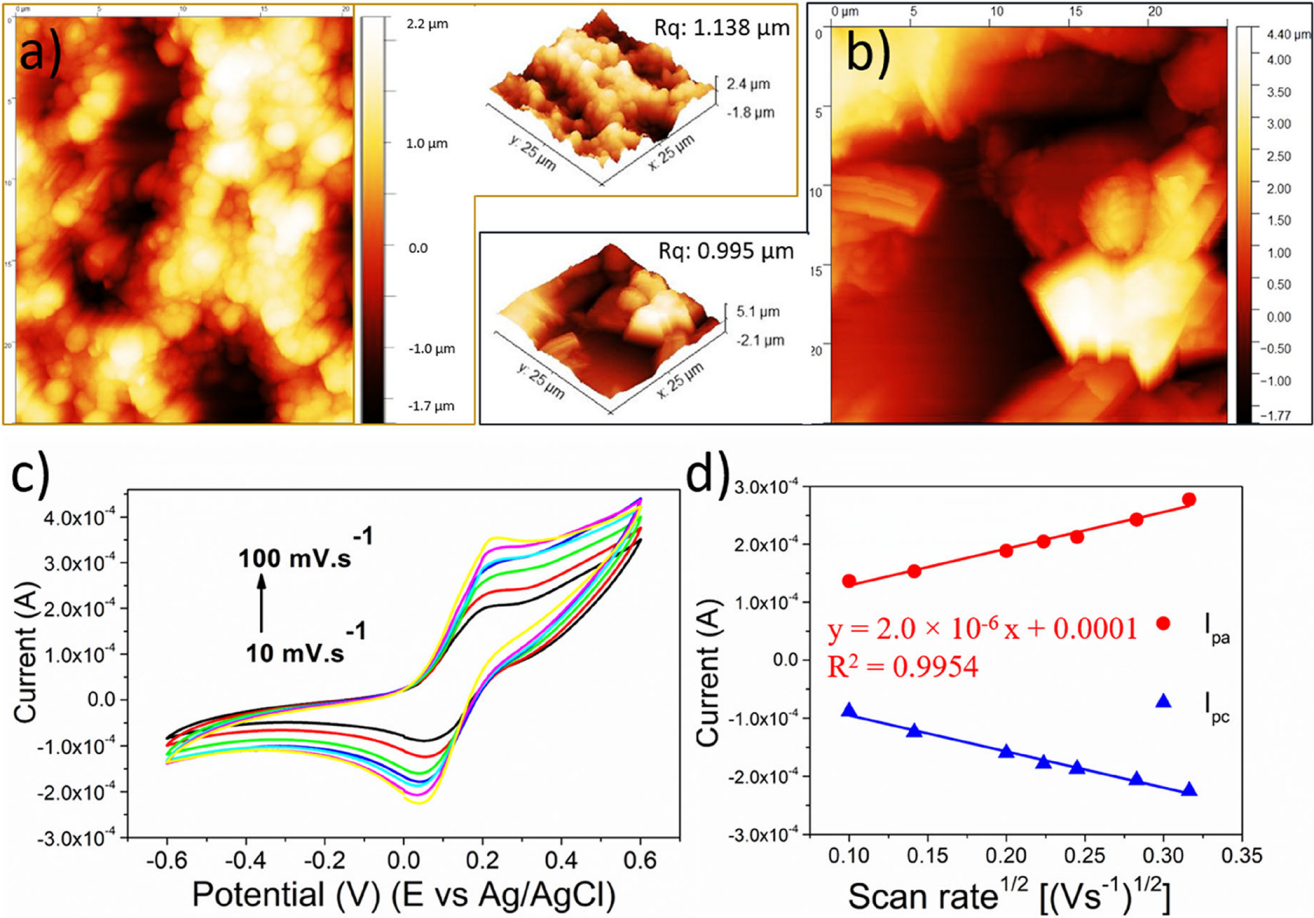
(a) AFM analyses of the Au film deposited in PCB. (b) AFM analyses of the Au film deposited in PCB with a biochar film. (c) Cyclic voltammograms of the developed sensor at different scan rates (10–100 mV·s^–1^). (d) Plotted peak currents for anodic and cathodic peaks vs the square root of the scan rate, including linear fits. All CVs were performed in the presence of K_3_[Fe­(CN)_6_]/K_4_[Fe­(CN)_6_] (10 mM) in NaNO_3_ (0.5 mol·L^–1^).

Glut, a dialdehyde, plays a pivotal role as a cross-linking agent, reacting with hydroxyl groups from biochar to form hemiacetal (reaction just with one hydroxyl group) or full acetal (when another hydroxyl group subsequently reacts). While the hemiacetal group is susceptible to hydrolysis, the latter is stable under neutral and acidic conditions.^60^ The formation of the full acetal assures the stability of the Glut-modified biochar; on the other hand, free aldehyde ends are also necessary to cross-link with the amine groups on the surface of the Cys-Au WE,^40,43^ acting as an anchoring system in the biochar.^27^ We conducted quantitative FTIR analyses with the utmost precision to investigate the formation of hemiacetal or full acetal structures under different Glut concentrations (ranging from 1.0 to 50%). The mass of the samples analyzed was weighed equally, and the results are shown in Figure 2. The broad bands observed between 3650 and 3100 cm^–1^ correspond to the O–H stretching vibrations, indicative of both inter- and intramolecular hydrogen bonding. The bands observed in 3000 and 2870 cm^–1^ are associated with C–H stretching from alkyl groups. The bands between 2720 and 2820 cm^–1^ are characteristic of the C–H axial vibration in the aldehyde group, CHO, while the peak at 1720 cm^–1^ confirms the presence of C=O stretching from the aldehyde group.^61,62^ The band around 1100 cm^–1^ is attributed to the C–O–C stretching, characteristic of acetal linkage.^63,64^ Additional bands observed are consistent with previously identified features of the biochar.

The cross-linker interaction between biochar and glutaraldehyde was evaluated in an initial analysis. As shown in Figure 2b, two main spectral features were examined. The first is the pronounced increase in the O–H stretching band in the 3650–3100 cm^–1^ region, indicating that gluta, when adsorbed to the biochar surface, promotes the formation of hydroxyls on the surface. This increase is observed progressively in samples with gluta concentrations ranging from 1 to 30%. However, at 50% concentration, a noticeable decrease in the intensity of the band of the O–H is observed. The second key feature is the band at 1100 cm^–1^, attributed to the C–O–C stretching. This band is observed in pure biochar and biochar/gluta samples. In pure biochar, it is attributed to the C–O–C stretching of aliphatic compounds inherent to the material.^65^ This same region in biochar/gluta samples also reflects the formation of acetal groups.^63,64^ Notably, in the modified samples, the band increases in intensity—indicating a greater presence of acetal groups—and shifts to a higher wavenumber. The shift to higher energy means a decrease in the length of the covalent bonds involved in the vibrational absorption. It may be associated with the cis or trans conformation of the C–O–C bonds of the acetal bridge between glutaraldehyde and biochar.^66,67^

Another important aspect to be analyzed is the distinction between the formation of hemiacetal and full acetal groups. As previously mentioned, hemiacetal groups are characterized by free aldehyde groups, which are capable of anchoring to the biochar surface and the electrode. The band at 1720 cm^–1^, characteristic of the C=O vibrational mode, is observed across all glutaraldehyde concentrations. However, the band at 2720 cm^–1^, indicative of the aldehyde group,^68^ is detected only in the Glut:50 sample. In addition, the Glut:50 sample exhibits a band in the 1700–1650 cm^–1^ region, which can be attributed to the formation of hemiacetals. The formation of hemiacetals occurs through the reaction between the carbonyl and hydroxyl groups from the acetal bridge on the opposite end of the glutaraldehyde molecule that already reacted with the biochar, as illustrated in Figure 2d. This interaction promotes an electronic delocalization or changes in the character of the carbonyl, thereby shifting the C=O band to a lower energy region.^69,70^ In our strategy, it is essential to achieve effective cross-linking between biochar and glutaraldehyde and the preservation of hemiacetal groups, leaving one aldehyde end available for anchoring to the Cys-Au electrode. The Glut:50 sample was chosen as the most suitable among these aspects with the addition of greater homogeneity and stability of the film.

Comparing the bare Au WE and the biochar-modified WE prepared with Glut 50%, the unmodified Au WE (Figure 3a, RMS (roughness mean square) = 1.138 μm) exhibited slightly higher roughness than the modified electrode (Figure 3b, RMS = 0.959 μm), indicating good surface uniformity and good homogeneity of the biochar-modified WE. The electrochemical behavior of the biochar sensors was evaluated by CV, as shown in Figure 3c. The electroactive surface area of the biochar-modified electrode was estimated to be 0.4593 mm^2^ using the Randles–Sevcik equation,^71^ based on CV measurements in a 10 mM [Fe­(CN)_6_]^3–^ solution containing 0.5 mol L^–1^ NaNO_3_.

$$i_{p}=2.69\times 10^{5}An^{3/2}D^{1/2}Cv^{1/2}$$ 2

where *i*_p_ is the peak current (A), *C* is the concentration of [Fe­(CN)_6_]^3–^ (mol·cm^–3^), ν is the scan rate (10–500 V·s^–1^), *A* is the electrode area (cm^2^), *n* is the number of electrons transferred (*n* = 1), and *D* is the diffusion coefficient (*D* = 7.6 × 10^–6^ cm^2^·s^–1^).^72^ From the CV results in Figure 3c, the anodic (*I*_pa_) and cathodic (*I*_pc_) peaks of the biochar sensor were identified. As the scan rate increased (Figure 3d), the anodic and cathodic peak potentials shifted toward more positive and more negative values, respectively. This behavior is characteristic of quasi-reversible electron transfer kinetics and supports the electrochemical activity of the biochar-modified sensor. A linear relationship was observed between the anodic peak current (*I*_pa_) and the square root of the scan rate, as described by the following regression equation:

$$I_{\mathrm{pa}}=2.0\times 10^{-6}x+0.0001$$ 3

This result suggests that the redox process is diffusion-controlled at the sensor interface. The EIS was applied to investigate the processes on the surface of the biochar-modified electrode and compare it with the bare electrode (Au WE). The measurements performed at the OCP are represented by the Nyquist plots, Figure S4a,b, and the Bode plot, Figure S4c. The bare sensor Nyquist plot showed a classic Randles circuit (Figure S4d), composed of a resistance, *R*_s_, related to the system components, the double-layer capacitance, *C*_dl_, in parallel with the charge transfer resistance, *R*_ct_, and the Warburg coefficient, *W*, related to the diffusion rate in the interface. On the other hand, the biochar-modified sensor showed an evident and huge semicircle in low frequencies (indicated by blue arrow) and a slight semicircle in high frequencies, evidenced in the inset in Figure S4b (indicated by black arrow). Due to the higher magnitude in the second semicircle, the first one is suppressed, but in the Bode plot (Figure S4c), the presence of different resistances in the system is evident. The Bode plot profiles are distinct, meaning that the equivalent circuits for bare and biochar sensors differ. Regarding the equivalent circuit of the systems, it is already known that the presence of a semicircle in the EIS data represents an element of a capacitor in parallel with a resistor. This element is also previously observed in the bare equivalent circuit represented by *C*_dl_ and *R*_ct_. Due to the two semicircles observed in the biochar sensor, it is assumed that there are two of these elements (Figure S4e). For the biochar sensor, it is speculated that the first capacitor and resistance elements are associated with the formation of the electric double layer and charge transfer resistance, respectively. Moreover, since biochar is a porous material with an irregular surface, the second element may be related to the diffusion of the probe through the bulk of the material, resulting in a higher resistance. Due to the characteristics of the biochar in the electrode, this behavior can be compared to what is observed in batteries and ceramic materials, where, at higher frequencies, resistance is attributed to ion mobility within the bulk of the grain.^73,74^

The heterogeneous electron transfer rate constant, *k* ^0^, was estimated for both the bare electrode (Figure S5) and the biochar-modified electrode (Figure 3c), using the Nicholson method^75^ for the [Fe­(CN)_6_]^3–^/[Fe­(CN)_6_]^4–^ redox couple, as described in eq 4:

$$k^{0}=\psi \sqrt{D_{O}\pi \nu nF/RT}$$ 4

where *D*_O_ is the diffusion coefficient (7.26 × 10^–6^ cm^2^·s^–1^), ν is the scan rate (V·s^–1^), *n* is the number of electrons involved in the process, *F* is the Faraday constant (C·mol^–1^), *T* is the temperature (K), *R* is the universal gas constant (J·K^–1^·mol^–1^), and ψ is a dimensionless kinetic parameter. Values of ψ = 0.44 and 0.1 were used for the unmodified and biochar-modified sensors, respectively, based on Δ*E* and the interpolation of Nicholson’s numerical data.^75,76^ The calculated *k* ^0^ values were 9.42 × 10^–4^ cm·s^–1^ for the bare electrode and 4.15 × 10^–3^ cm·s^–1^ for the biochar-modified electrode. These results demonstrate that the biochar modification enhances electron transfer kinetics, enabling a faster and more sensitive response than the unmodified PCB-Au electrode.^25^

### 3.3 Anti-cTnT Immobilization and Characterization

The anticardiac Troponin T antibody was immobilized onto the biochar electrode, adding 20 mM Cys and Glut. Cys was used as a cross-linking agent, facilitating the interaction between the carbonyl (C=O) and hydroxyl (O–H) groups on the Glut-modified biochar electrode with the aldehyde groups from 50% Glut. These aldehyde groups are highly reactive and capable of forming covalent bonds with the amine groups of the cTnT antibody, ensuring stable immobilization. Confocal fluorescence micrographs were obtained before and after immobilizing the anticardiac Troponin T antibody onto the biochar electrode (cTnT immunosensor). Fluorescence analyses without antibody immobilization (Figure 4a) show low intensity and emission bands centered around 600–650, 660, and 700 nm (Figure 4a inset). Emissions at these longer wavelengths (in the red region) are generally associated with molecules containing more conjugated bonds, such as aromatic rings.^77,78^ This observation is consistent with aromatic structures previously identified in the biochar material (Figure 1).

**Figure 4:**
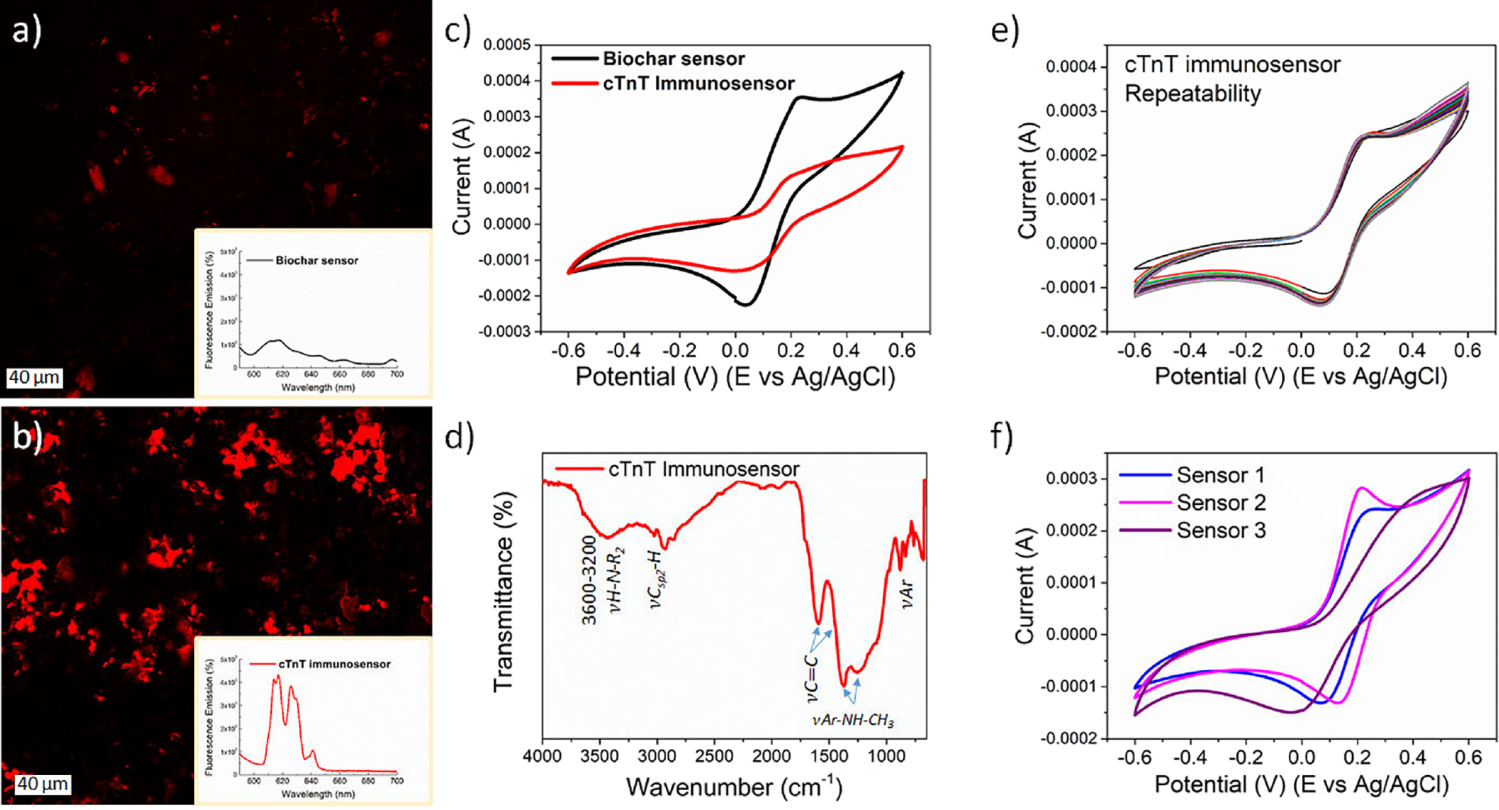
(a) Fluorescence Confocal Micrographics of a biochar-based sensor board. (b) Fluorescence Confocal Micrographics of a biochar sensor board with cTnT antibody immobilization. All sensors were incubated with fluorescent secondary antibody. (c) CVs obtained for the Biochar-based sensor board (black) and cTnT immunosensor (red). (d) FTIR analysis for Biochar-based sensor board (green) and cTnT immunosensor (blue). (e) 20 consecutive cTnT immunosensor scans. (f) CV of three different cTnT immunosensors. All CVs were performed in the presence of K_3_[Fe­(CN)_6_]/ K_4_[Fe­(CN)_6_] (10 mM) in NaNO_3_ (0.5 mol·L^–1^), with a scan rate of 100 mV.s^–1^.

In the fluorescence analysis after the immobilization of the cTnT immunosensor (Figure 4a,b), a significant increase in the emission intensity is observed in the micrograph and the corresponding spectroscopic data (Figure 4a,b inset). The spectra show intense emission bands in the 610–630 nm range, with lower-intensity bands near 640 nm. These emissions correspond to orange and red regions of the visible spectrum, respectively. According to the literature, antigens, antibodies, and their immobilized complexes exhibit fluorescence at different wavelengths. Green emissions are generally associated with the protein components of antibodies; red emissions correspond to molecules with many bonds π such as polysaccharides, lipids and proteins, reflecting the composition of antigens; while orange emissions are typically attributed to the specific interaction between antigen epitopes and antibody paratopes.^79–81^ Therefore, the intense emission in the orange region (610–630 nm) provides strong evidence of oriented immobilization of the anticardiac Troponin T antibody onto the biochar surface through the strategy shown in this paper using a higher concentration of Glut and Cys.

The immobilization efficiency of the anticardiac Troponin T antibody onto the biochar surface is also analyzed by CV and FTIR. A decrease in the *I*_pa_ of the cTnT sensor is observed compared to the board sensor without anti-cTnT antibody immobilization (Figure 4c). The reduction of the *I*_pa_ occurs due to the isolating characteristics of the antibody. The spectrum obtained by FTIR (Figure 4d) shows the presence of a medium-strong band in the region of 3400 cm^–1^, which characterizes secondary amine stretching.^82^ The weak band at 3022 cm^–1^ is characteristic of C_sp2_–H stretching of aromatic rings, as confirmed by bands at 886, 833, 757, and 687 cm^–1^, indicative of disubstituted aromatic rings. The band at 1594 cm^–1^ and the shoulder of the band at 1440 cm^–1^ refer to C=C stretching of the aryl group. The bands at 1380 and 1240 cm^–1^ are characteristic of aryl alkyl amine groups. Therefore, these bands are characteristics of amine groups and are associated with antibody immobilization. EDS analysis also shows the nitrogen (N) presence in the cTnT immunosensor, which corroborates previous data showing a homogeneous distribution of the cTnT antibody on the biochar WE surface, proving that antibody immobilization occurred on biochar WE (Figure S6).

The cTnT immunosensor’s repeatability was tested by performing 20 consecutive voltammograms (Figure 4e). The immunosensor showed excellent stability, with a CV of 1.879%. cTnT immunosensor reproducibility was also investigated. The data obtained by cyclic voltammetry from the triplicate of the immunosensors showed a CV of 9.6% (Figure 4f), indicating good reproducibility of the method developed here to prepare electrochemical biosensors from biomass.

### 3.4 Immunosensor Response to cTnT

Another crucial aspect in the development of electrochemical immunosensors is the ability of the antibody to bind specifically to its target antigen. To assess this, we evaluated the specificity of the antibody using dot blot analysis (Figure 5a). The dot blot assay is a qualitative immunological technique used to confirm the selective recognition of an antibody for its corresponding antigen. The results demonstrated that the antibody successfully binds to the cTnT antigen in a concentration-dependent manner. The assay was sensitive enough to detect cTnT concentrations as low as 15 ng, indicating good specificity and binding affinity of the antibody under the tested conditions.

**Figure 5:**
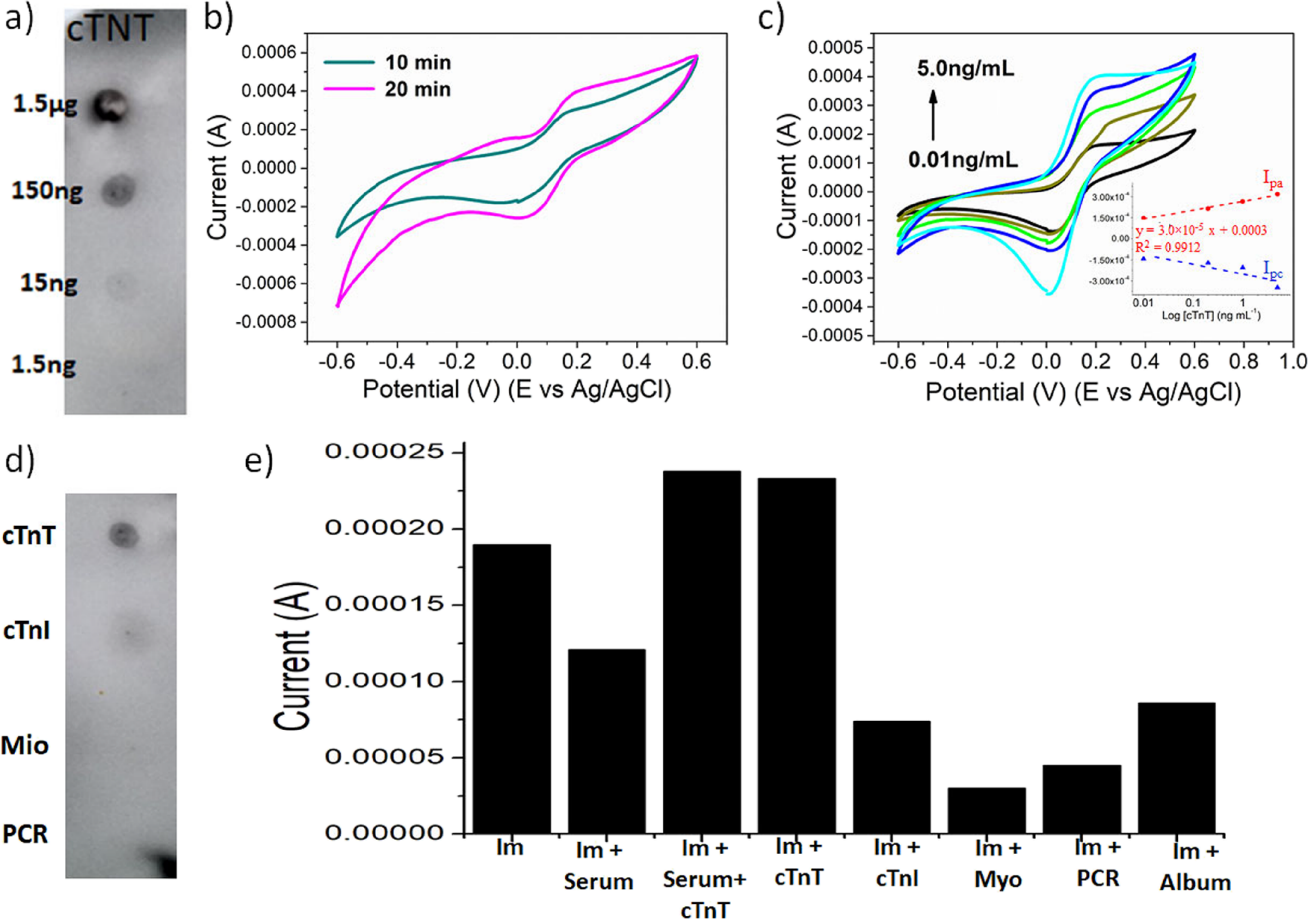
(a) Dot blot for cTnT antigen in different concentrations incubated with anti-cTnT (1:5000). (b) Voltammograms obtained from CV analyses of cTnT immunosensors incubated with cTnT (0.1 ng·mL^–1^) for 10 min (gray) and 20 min (pink). (c) Voltammograms obtained from CV analyses of cTnT immunosensors incubated with different cTnT isolated proteins (inset: linearity curve). (d) Dot blot using cTnT, cTnI, Myo, and CRP incubated with antibody anti-cTnT (1:5000). (e) Bar graph representing the selectivity results of the immunosensor against different biomolecules.

Different incubation times (10 and 20 min) of cTnT (0.1 ng·mL^–1^) were tested to determine the optimum interaction time between the antibody and antigen. A higher anodic peak was observed after 20 min of incubation (Figure 5b). Therefore, 20 min was established as the optimal incubation time for accurate detection of cTnT. This time is significantly shorter than 1 h, which is typically recommended for cTnT evaluation using disposable tests. The rapid detection of cTnT is critical for the early diagnosis of AMI, as irreversible cardiac tissue damage due to necrosis can occur approximately 6 h after the onset of AMI symptoms.^47,48^

CV analyses obtained the calibration curve of the electrochemical immunosensor (Figure 5c). The anodic peak increases with an increasing cTnT concentration, indicating that the binding of the recombinant cTnT protein with the anti-cTnT antibody occurs through the release of the electrons. In the sensing mechanism, the oxygen species present on the surface of the biomass bind to cys and provide amino groups on their surface. These groups bind to glut, aiming to get carbonyl groups easily bound to amino groups from the anti-cTnT molecule. Finally, the cTnT epitope in the antibody is free of binding to the cTnT recombinant. After binding, recombinant cTnT releases electrons assisting to reduce [Fe­(CN)_6_]^3–^ into [Fe­(CN)_6_]^4–^. The [Fe­(CN)_6_]^4–^ ions are again oxidized to [Fe­(CN)_6_]^3–^ during the forward CV scan, and the transfer of electrons occurs, leading to an increase in *I*_pa_ corresponding to an increase in recombinant cTnT. The immunosensors show a linear and stable response between 0.01 and 5.0 ng·mL^–1^ of the cTnT with a correlation coefficient (*r* ^2^) equal to 0.991, indicating a strong positive linear relationship between the concentration of cTnT and the response of the immunosensor, making it an excellent test to detect cTnT, Figure 5c inset. The limit of detection (LOD) was calculated according to the eq 5:

LOD=S+3×SD 5

where *S* is the mean value of the signal for a blank measured multiple times, and SD is the standard deviation.^45^ We found an LOD of 0.003 ng·mL^–1^. This low LOD allows the immunosensor to detect cTnT in healthy individuals and patients experiencing AMI. Clinically, serum cTnT concentrations of approximately 0.014 ng·mL^–1^ are typical in healthy individuals. In comparison, levels exceeding 0.050 ng·mL^–1^ indicate ongoing AMI, making it a precise point-of-care test. Our results were compared with recent cTnT detection research, where we found a lower LOD using a printed circuit board-based sensor and a sustainable material. This comparison also highlights the novelty of our findings (Table 1).

**Table 1:** Comparison of the Analytical Performance of the Developed Immunosensor with Recent Tests for the Diagnosis of cTnT^a^

method	immobilization method	LOD	TnT incubation time (min)	linear range (ng mL^–1^)	ref
EC (DPV and CV)	cTnT immobilization on biochar through a cross-link reaction mechanism via glutaraldehyde	0.003 ng·mL^–1^	20	0.01–5.0	this work
EC (EIS and CV)	graphite paper electrodes incubated with EDC/NHS pair as a cross-linker	1.28 fg·mL^–1^	30	5.0 × 10^–7^–0.001	^19^
EC (EIS and CV)	a 3D hierarchical nanoarchitecture immunosensor using AuNPs/MXene@PAMAM	0.069 ng·mL^–1^	40	0.1–1000	^83^
EC (SWV and CV)	immunosensor based on a nanohybrid film of carboxylated polypyrrole and amine nanoclay	0.35 pg·mL^–1^	60	0.0025–0.125	^84^
Optical (SPR)	SPR method-based immunosensor using AuNPs-anti-cTnT	0.5 ng·mL^–1^	3	0.5–40	^85^
Optical (SPR)	a SAM of 1-octanethiol and (11-MUA#) formed on an annealed Au thin film modified with EDC/NHS for amine coupling to cTnT	0.0625 μg·mL^–1^	12–15	62.5–1000	^86^

a EC: Electrochemical; SPR: surface plasmon resonance; SAM: self-assembled monolayer. #11-MUA: 11-mercaptoundecanoic acid.

### 3.5 Selectivity and Recovery of the Immunosensor

Selectivity is a feature that demonstrates that the immunosensor can bind only to cTnT, even in the presence of several compounds. Selectivity was evaluated by incubating the cTnT immunosensor with fetal bovine serum (FBS) to simulate human serum, with and without spiked with cTnT (0.1 ng·mL^–1^) and albumin (BSA 3.5 g·dL^–1^). Albumin was also analyzed because it is the main protein present in human serum. The assay in the presence of the antigen was realized in duplicate to confirm the reproducibility of the sensor even in the presence of interferences. CV and DPV results, Figures 5e and S6, show a decrease of the *I*_pa_ when the immunosensor is incubated with pure bovine serum or albumin due to their insulating characteristics. However, the *I*_pa_ increases for the samples spiked with antigen, demonstrating that the interaction between the antigen and the anti-cTnT antibody occurs even in a complex matrix. Dot blot analysis further confirmed the selectivity of the anti-cTnT antibody, demonstrating strong binding to the cTnT antigen and no cross-reactivity with structurally similar or common serum proteins, such as cardiac troponin I (cTnI), C-reactive protein (CRP), or myoglobin (Figure 5d). These findings were corroborated by electrochemical measurements, where no significant increase in *I*_pa_ was observed when the immunosensor was incubated with cTnI, CRP, or myoglobin (Figure S6b), reinforcing the specificity of the sensor for cTnT. We must highlight the unique advantage of using undiluted serum in our analyses. It reduces sample handling steps, cost, and contamination risk, giving the point-of-care tests developed from biomass-based sensors a significant edge.

The recovery evaluation of a biosensor demonstrates how much of the target molecule can be detected in the presence of a complex matrix such as human serum. Analyzing the data from the immunosensor in the presence of 0.1 ng·mL^–1^ cTnT and the serum spiked with 0.1 ng·mL^–1^ cTnT (Figure 5e), we observe an *I*_pa_ of 2.335 × 10^–4^ A for pure cTnT and 2.377 × 10^–4^ A in the presence of serum. Based on these data, we calculated the recovery using eq 6. A recovery of 102% was found in the presence of fetal bovine serum (Table S1), a clear indication of the successful recovery of our immunosensor in a complex matrix. This finding, although based on a single concentration, underscores the effectiveness of our immunosensor in detecting the protein in a complex matrix at a concentration of 0.1 ng·mL^–1^.

Recovery%=(amount⁢ of⁢ analyte⁢ recovered/amount⁢ ⁢of⁢ ⁢analyte⁢ added)x100 6

### 3.6 Bare Board Sensor Reuse

Many researchers in recent years have pursued environmentally friendly, nonpolluting, with minor waste generation to be used in devices to meet the circular economy requirements. It is based on sharing, recycling, repairing, and recycling materials and products. The cTnT immunosensor prepared here uses biochar from cassava biomass to aid in the immobilization of the antibody and improve the path of the electrons in the working electrode. The methodology to obtain biochar from cassava undergoes heat treatment without adding chemical compounds, making it eco-friendly. This biomass is abundant in nature and has a closed life cycle. Using cassava waste to make the immunosensor meets one of the prerequisites of a circular economy.

In addition, we also verified the possibility of reusing/repairing/recycling the bare board sensor made in PCB to make it last longer, another prerequisite of a product to meet the circular economy. After its first use, we tested its reuse by washing the bare board sensor with deionized water to remove the biochar and antibodies present on its surface. After cleaning, the bare board sensor was dried with nitrogen, and the immunosensor was reprepared for reusing. Figure 6 shows no significant change in *I*_pa_ value even after three times reusing the bare board sensor, demonstrating the possibility of recycling, repairing, and reusing it at least three times. Another advantage of using PCBs is that they can also be recycled, helping reduce electronic waste generation, decreasing the demand for raw material extraction, and lowering the electronic sector’s carbon footprint.

**Figure 6:**
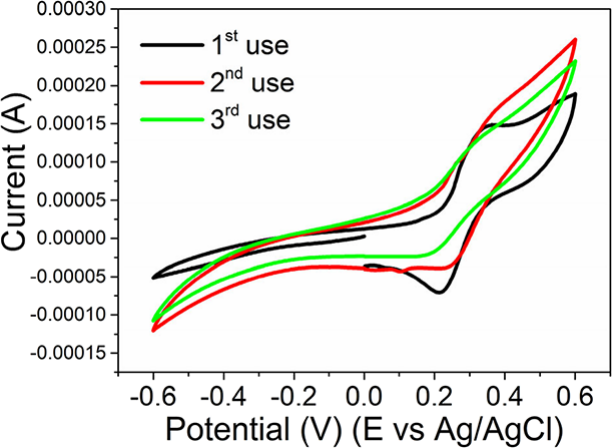
CVs obtained in the first use of the immunosensor (black), after the second use (red), and after the third use (green). All CVs performed in the presence of K_3_[Fe­(CN)_6_]/K_4_[Fe­(CN)_6_] (10 mM) in NaNO_3_ (0.5 mol·L^–1^), with a scan rate of 100 mV·s^–1^.

## 4 Conclusion

An electrochemical immunosensor based on biochar surface functionalization with glutaraldehyde was successfully developed for application as a rapid and portable immunosensor for cTnT detection. The biochar was synthesized through the pyrolysis of cassava biomass, and it is composed of micrometric and porous particles with a molecular-level structure rich in oxygen bridges. These features make it suitable for surface functionalization and lead to increased electrochemical responses with better electron transfer kinetics. Our study evaluated the concentration and molecular interaction conditions of glutaraldehyde-biochar to determine the optimal ratio for producing homogeneous and stable films for biosensor development. The concentration of the Glut solution used during the preparation of the biochar electrode influenced the obtention of homogeneous biochar films and the quality and stability of the immunosensor. The optimal ratio identified was Glut 50%: 0.01g biochar. This proportion favored the formation of hemiacetal structures, retaining one free aldehyde group capable of covalently anchoring to the Cys-modified gold working electrode. The free aldehyde group from the hemiacetal enabled effective binding with the Cys-Au WE, resulting in a more uniform and mechanically stable biochar film, which is essential for reproducible sensor fabrication and reliable analytical performance. Immunofluorescence confocal microscopy, VC, FTIR, and EDS analyses proved efficient immobilization of the anti-cTnT antibody onto the biochar surface. The electrochemical cTnT immunosensor prepared showed high sensitivity, with a detection capacity of 0.01–5.00 ng·mL^–1^, a LOD of 0.003 ng·mL^–1^, and high selectivity in albumin and bovine serum samples. The biochar-based immunosensor is an eco-friendly, portable, and easy-to-handle tool for point-of-care diagnosis of AMI. There was no significant change in *I*_pa_ value even after three times reusing the bare board sensor, demonstrating the possibility of recycling, repairing, and reusing it. The results shown in this work open the way for using biochar on PCB in new biosensor devices that meet circular economy requirements.

## Supplementary Material



## Notes

SEM analyses of the biochar powder at various magnifications, BET analysis for the biochar, SEM analyses of biochar-based board sensors, impedance data of biochar and immunosensors, cyclic voltammograms of the PCB, EDS analysis of the cTnT immunosensor, selectivity analyses for CV from the immunosensor, and recovery analyses (PDF)
