# Supplementary material for: Surface Modification of Biochar to Prepare Environmentally Friendly Electrochemical Biosensors for Detection of Cardiac Troponin T
Source: ACS Omega. 2025 Jun 9;10(24):25842–54. doi: 10.1021/acsomega.5c02113 (PMC12199043; doi:10.1021/acsomega.5c02113)
Supplement: Supplementary file 1 [file ao5c02113_si_001.pdf]

SUPPLEMENTARY INFORMATION

Surface Modification of biochar to prepare environmentally friendly electrochemical biosensors for detection of cardiac troponin T

*Aline Macedo Faria[1], Rafael Aparecido Ciola Amoresi[2], Larissa Bach-Toledo[1], Juan Andrés[2], Talita Mazon[1]**

[1] Centro de Tecnologia da Informação Renato Archer (CTI) - Ministério da Ciência, Tecnologia e Inovação (MCTI), Rod. D. Pedro I, KM 143.6, 13069-901 Campinas, SP, Brazil

[2] Department of Analytical and Physical Chemistry, University Jaume I (UJI), Av. Vicent Sos Baynat, 12071, Castelló, Spain

* Corresponding author: e-mail: talita.mazon@cti.gov.br

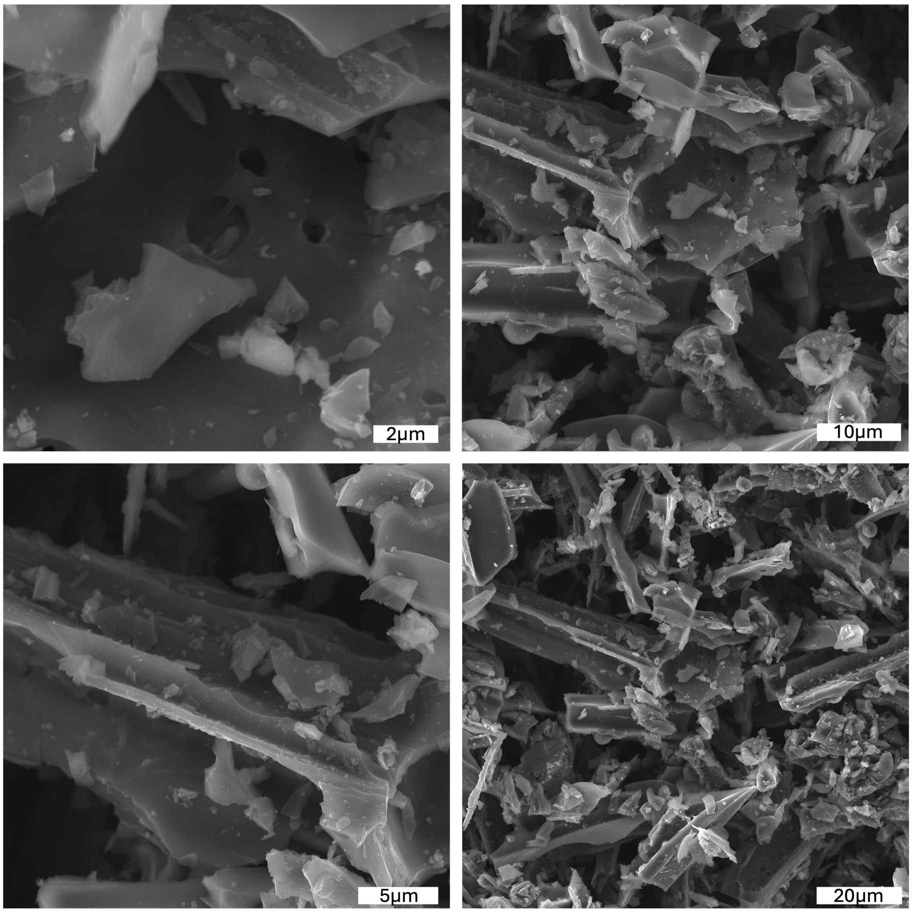

Figure S1. SEM analyses of the biochar powder at various magnifications.

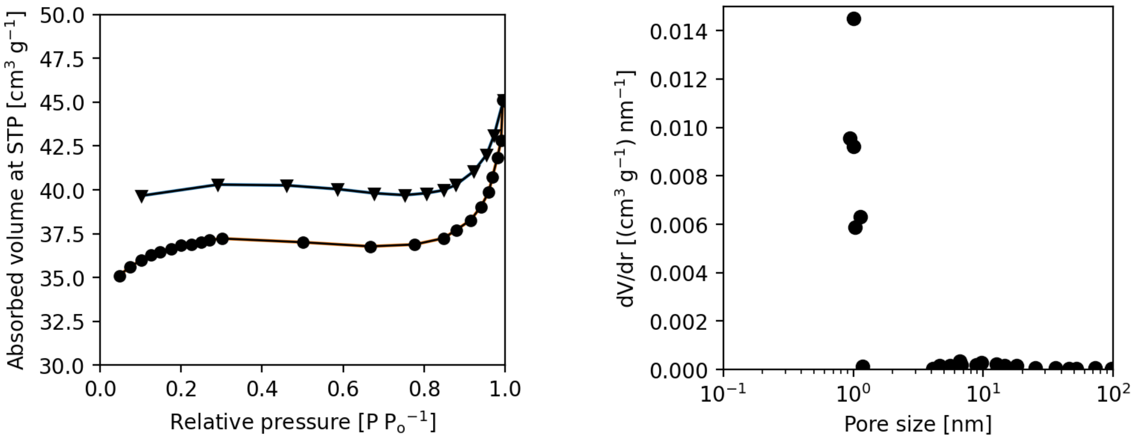

Figure S2. (a) Nitrogen adsorption and desorption isotherms at 77 K and **(b)** BJH pore size distribution for the biochar.

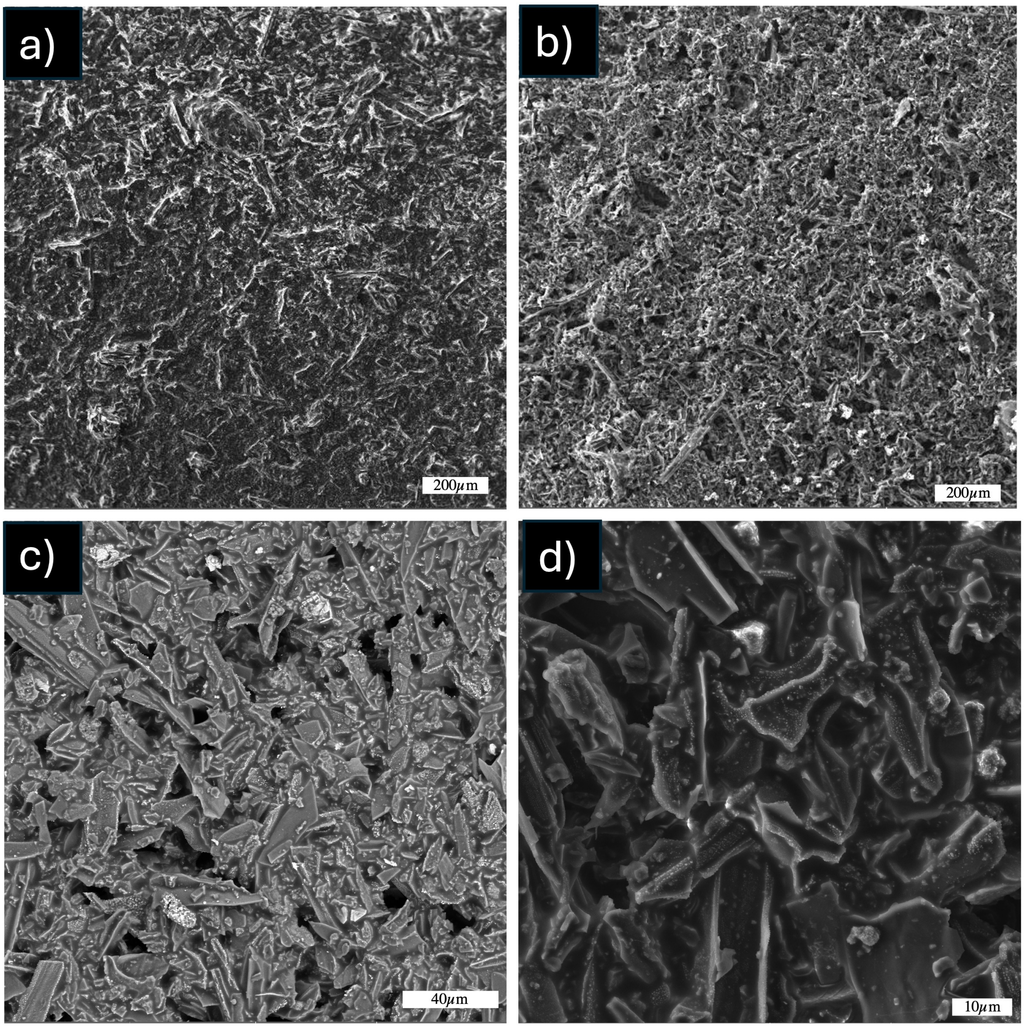

Figure S3. SEM analyses. (a) Biochar-based board sensor prepared with Glut-2.5%. (b-d) Biochar-based board sensor prepared with Glut-50%, with electrons secondary mode (b and d) and backscattering mode (c).

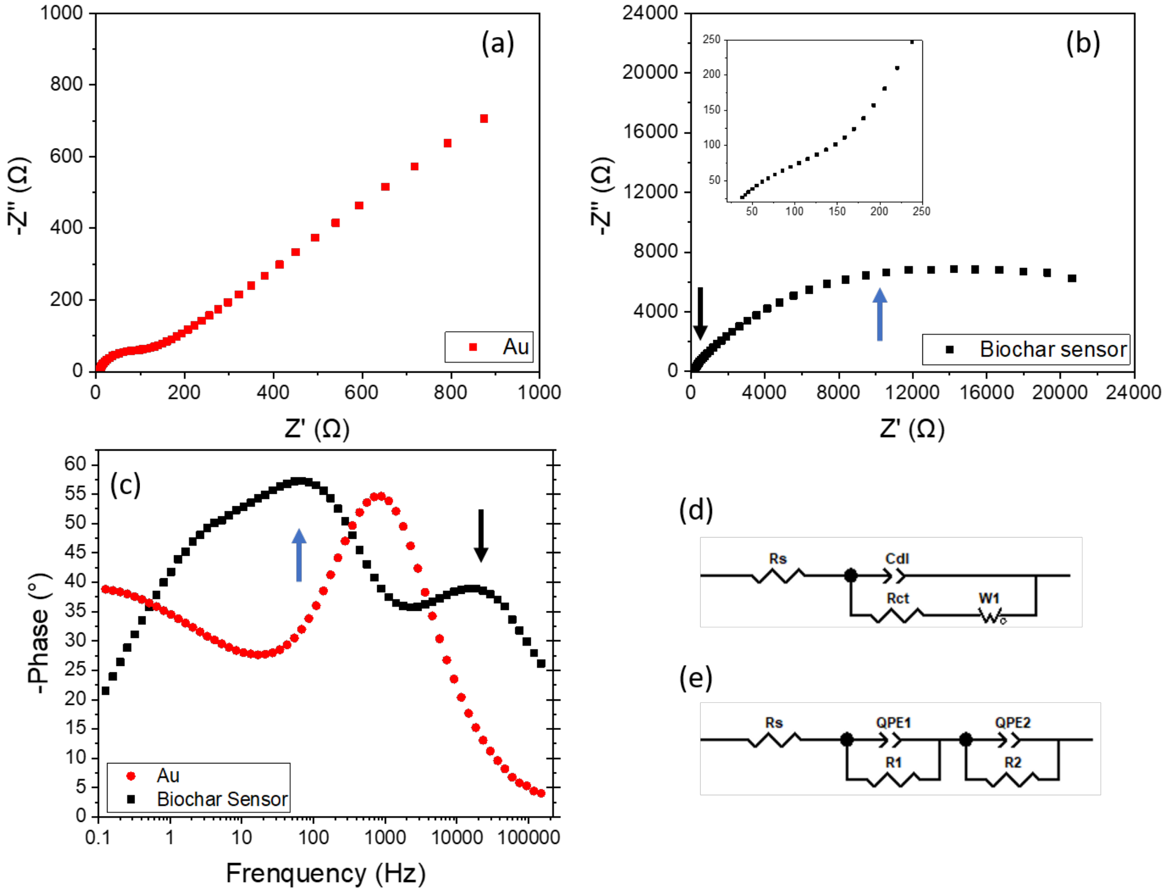

Figure S4. EIS data. Nyquist plots for (a) bare and (b) biochar sensors. (b) Bode plots represent the phase angle as a function of frequency for both sensors. An equivalent circuit is proposed for (d) bare and (e) biochar sensors. Regions with low and high frequencies are indicated by blue and black arrows, respectively.

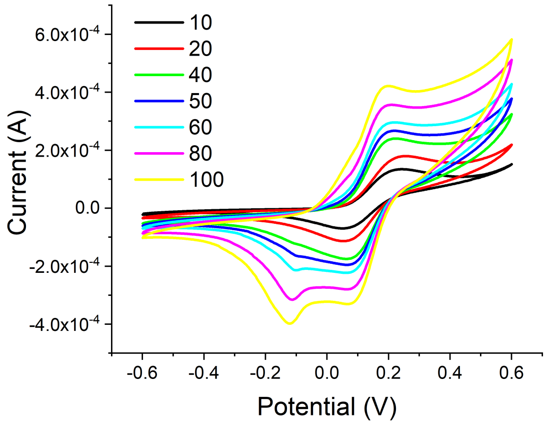

Figure S5. Cyclic voltammograms of the PCB-Au electrode at different scan rates (10 -100 mV s^{-1}), performed in the presence of $K_3[Fe(CN)_6]$/ $K_4[Fe(CN)_6]$ (10mM) in $NaNO_3$ (0.5 mol L^{-1}).

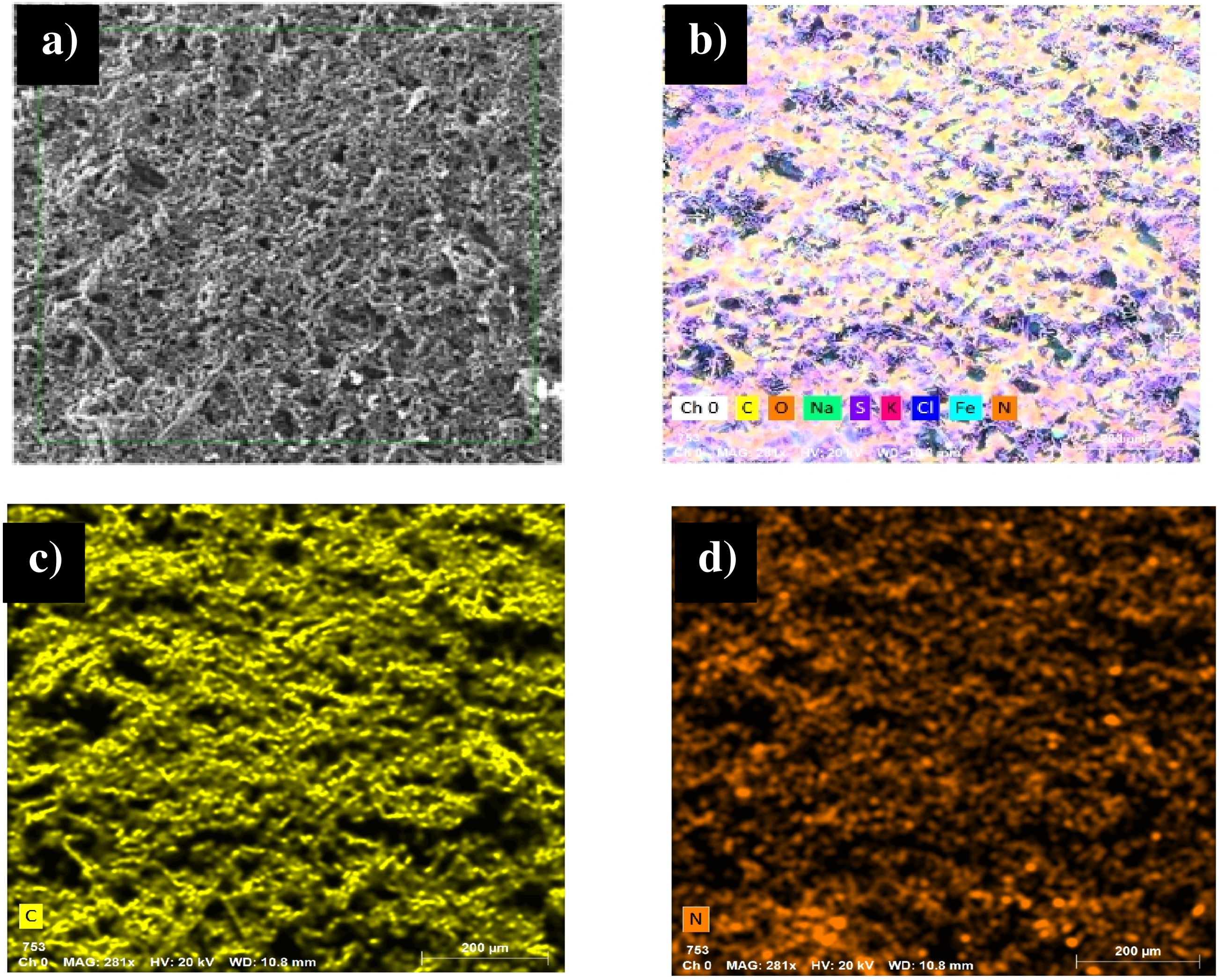

Figure S6. EDS analyis of cTnT immunosensor. A) cTnT immunosensorm micrography. b) Presence of Ch in white, C in yellow, O in orange, Na in green, S in purple, K in pink Cl in blue, Fe in Cian and N in light orange. c) Carbon distribution in cTnT immunosensor. d) Nitrogen distribution in cTnT immunosensor.

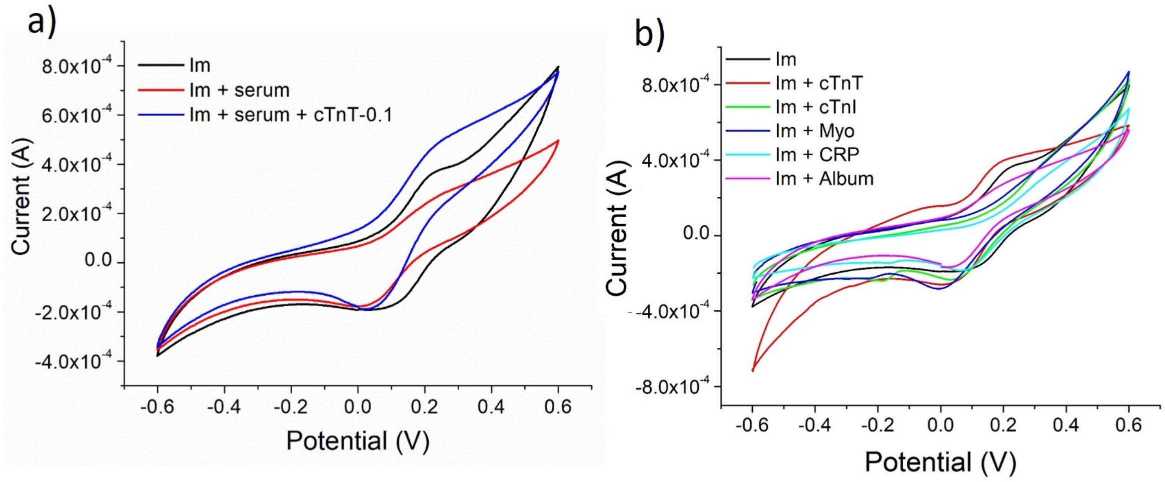

Figure S7. (d) CV analyses for the immunosensor (black), immunosensor incubated with serum (red), and immunosensor incubated with serum plus cTnT (0.1ng mL^{-1}) (blue) (e) CV analyses for the immunosensor (black), immunosensor incubated with serum (red), and immunosensor incubated with cTnT (red), immunosensor incubated with cTnI (green), immunosensor incubated with Myo (dark blue), immunosensor incubated with PCR (blue) and immunosensor incubated with the albumin (pink). All the CVs were performed in the presence of $K_3[Fe(CN)_6]$/ $K_4[Fe(CN)_6]$ (10mM) in $NaNO_3$ (0.5 mol L^{-1}), with a scan rate of 100mV s^{-1}.

Table S1. Recovery found for 0.1ng.mL^{-1} cTnT in the presence of fetal bovine serum.

cTnT added (ng.mL^{-1})	cTnT found (ng.mL^{-1})	Recovery (%)
0.1	0.1	102
